# Brixia score on initial chest X-ray as a predictor of outcome in COVID-19 and H1N1 acute chest infections: A retrospective study

**DOI:** 10.1097/MD.0000000000047295

**Published:** 2026-01-23

**Authors:** Ruba Khasawneh, Basheer Khassawneh, Firas A. Khasawneh, Maha Gharaibeh, Sarah Athamneh, Tarek Ajam, Mohammad Z. Nofal

**Affiliations:** a Department of Diagnostic Radiology and Nuclear Medicine, King Abdullah University Hospital, Jordan University of Science and Technology, Irbid, Jordan; b Department of Internal Medicine, King Abdullah University Hospital, Jordan University of Science and Technology, Irbid, Jordan; c Department of Mechanical Engineering, Michigan State University, East Lansing, MI; d Department of Internal Medicine, Hamad Medical Corporation, Doha, Qatar.

**Keywords:** Brixia score, chest X-ray, COVID-19, disease severity, H1N1, prognosis, radiographic scoring system

## Abstract

This study aims to evaluate the use of the Brixia score on chest X-ray to predict disease severity, progression, and outcomes across 2 distinct viral infections: COVID-19 and H1N1. A retrospective analysis was conducted on patients diagnosed with COVID-19 (March 1, 2020–January 1, 2021) and H1N1 (January 1, 2016–January 1, 2021) at a tertiary hospital. Chest X-rays obtained within 24 hours of presentation were scored using the Brixia score (score at admission) by 2 board-eligible radiology residents. Scoring of subsequent images was also performed to assess disease progression by documenting the highest score, lowest score, and score at death or discharge. This study included 953 COVID-19 patients and 222 H1N1 patients. The number of male cases was predominant in both COVID-19 (56.5%) and H1N1 (53.2%). The median age was higher in COVID-19 patients (60 years) compared to HIN1 patients (40 years) (*P* < .001). COVID-19 patients had a higher mortality rate and greater severity as depicted by Brixia scores at the time of admission (8.35 vs 6.77) and at discharge/death (12.64 vs 12.33; *P* < .001). Hypertension was the most common comorbidity in COVID-19 patients (57.3%) and HN1 patients (26.1%) (*P* < .001). Our study demonstrates the prognostic value of the Brixia score in predicting outcomes across COVID-19 and H1N1 patients. COVID-19 patients had higher Brixia scores and greater score increments over time, correlating with increased mortality. These findings demonstrate the role of chest radiographic assessment in early risk determination and clinical decision-making.

## 1. Introduction

Numerous contagious viral pandemics have been encountered in the early 21st century,^[1]^ such as the swine-origin influenza A (H1N1) pandemic in 2009 and the more recent coronavirus pandemic affecting 60.8 million people and yielding 284,000 deaths worldwide.^[2]^ On January 6, 2020, a novel Coronavirus was revealed after a group of patients had viral pneumonia of unknown origin in Wuhan, China. It was termed “severe acute respiratory syndrome coronavirus 2”. Most patients were exposed to the Huanan Seafood Market in the same city.^[3]^ This virus has led to about (774,771,942) confirmed cases and (7035,337) deaths worldwide.^[4]^

Several studies have investigated the imaging features of those viral illnesses.^[1–6]^ Most of the studies focused on their computed tomography [CT] imaging appearance. Bai et al showed that imaging characteristics had a high specificity but moderate sensitivity in differentiating COVID-19 pneumonia from other viral pneumonia.^[7]^

A chest X-ray (CXR) scoring system serves as a semi-quantitative system utilized by radiologists to evaluate the severity of lung pathologies including acute chest infections. Several CXR scoring systems were suggested during the latest COVID-19 pandemic, to stratify patients according to illness severity. The most widespread CXR scoring system is the Radiographic Assessment of Lung Edema score,^[8,9]^ which was then adapted for the assessment of viral pneumonias by assessing the number of CXR opacities in each lung zone. One of the commonly used scoring systems for COVID-19 is the COVID-19 Reporting and Data System (CO-RADS); however, it was based on CT scan findings.^[10]^ One of the major advantages of CXR is their speed and affordability compared to other imaging techniques which makes them a practical choice for rapid assessment.^[11]^ The Brixia score was initially developed in 2015 for non-radiologist clinicians.^[12]^ The Brixia scoring system evaluates CXR findings and quantifies the extent of lung parenchymal involvement. During the COVID pandemic, it was 1st implemented in Italy to assess the severity of COVID-19 infection.^[13,14]^ Subsequently, several studies have directly compared the utility and performance of the Brixia score compared to other CXR scoring systems.^[15,16]^ Another study has assessed its performance in predicting COVID-19-related thrombo embolisms.^[17]^ Many other studies have correlated the CXR scoring systems with different clinical parameters for predicting COVID-19 disease severity.^[18,19]^ A CXR scoring system modified from the Brixia score has been adopted in some studies to provide insights about the predictive value of this scoring system in determining the clinical course and prognosis of patients with COVID-19 in terms of their outcome based on noninvasive ventilation, intubation or death.^[20]^ In a more recent study, Tenda et al^[21]^ compared the performance of the Brixia score with an AI scoring system for predicting the severity of COVID-19 pneumonia.

This article aims to assess the Brixia score at initial CXRs (score at admission [A-score]) and the subsequent scores during hospital stay in COVID-19 and H1N1 and to correlate them with patients’ age, gender, comorbidities, as well as patient outcome. To the best of our knowledge, this is the 1st study to use both COVID-19 and H1N1 patient cohorts to evaluate the Brixia score’s performance across distinct viral pneumonias.

## 2. Materials and methods

### 2.1. Study design

The study employed a retrospective design conducted at a tertiary hospital. Patient data were retrieved from the hospital’s medical record system (iSOFT) by reviewing records of patients diagnosed with COVID-19 and H1N1 confirmed through reverse transcription polymerase chain reaction testing of nasopharyngeal swab specimens.

### 2.2. Sampling procedure

**Figure 1.**
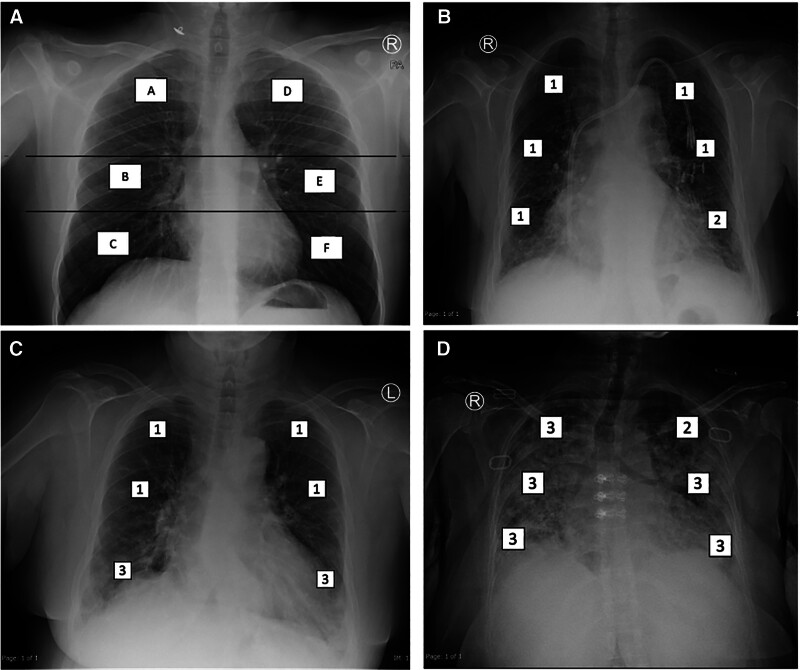
(A) Frontal chest X-ray demonstrating the levels (horizontal lines) and zonal anatomy (A–F) of the Brixia score; (B) frontal chest X-ray for a 77-year-old female at admission due to H1N1, demonstrating the Brixia score with interstitial predominance; (C) frontal chest X-ray in COVID-19 male patient: predominantly interstitial markings in lungs; (D) frontal chest X-ray in COVID-19 male patient: predominantly interstitial markings in lungs at admission; (E) frontal chest X-ray in COVID-19 male patient: predominantly alveolar infiltrates bilaterally; (F) frontal chest X-ray in H1N1 female patient: predominantly alveolar.

The included dates for COVID-19 patients were from March 1, 2020 to January 1, 2021 while for H1N1 were from January 1, 2016 to January 1, 2021. All patients with at least 1 frontal CXR obtained within 24 hours of their presentation regardless of age were included in this study. The CXR at admission was scored using the Brixia score (A-score)^[14]^ by 2 board-eligible radiology residents who were blinded to patients’ details and then confirmed by a board-certified radiologist with 13 years’ experience. This score divides each lung into 3 zones as demonstrated (Fig. 1). Each zone is then scored according to the parenchymal findings as follows: (0) score corresponds to normal, (1) corresponds to interstitial disease, (2) corresponds to interstitial and alveolar opacities with interstitial predominance, while (3) corresponds to interstitial and alveolar opacities with an alveolar predominance. The scores from each lung zone were summed up to generate a total severity score ranging from 0 to 18. The patients were further categorized according to Brixia score disease severity as follows: 0 score is considered normal, a score between 1 and 6 corresponds to mild disease, a score between 7 and 12 is defined as moderate disease, while a score between 13 and 18 is defined as a severe disease. This was based on available literature. Subsequent CXRs for admitted patients were also examined and compared with the initial CXR to document the highest score (H-score), lowest score (L-score), and the score at death or discharge (D-score). For patients with only 1 CXR, the assessment was based solely on the A-score. Other CXR findings assessed included: pleural effusion, pneumomediastinum, pneumothorax, and lung fibrosis categorized as present or absent according to defined criteria. Lung fibrosis was defined according to the Fleischner Society Glossary^[22]^; as the presence of reticulations and honeycombing with associated ground glass opacities and tractional bronchiectasis. Patient demographics (age, and sex), and clinical outcome (defined as death or discharge) were also retrieved from the iSOFT and correlated with the CXR severity score.

### 2.3. Data analysis

Statistical Package for Social Sciences (SPSS) version 27 (IBM Corp., Armonk)^[23]^ was used for statistical analysis and management of research data. The statistical analyses employed in this research include frequency distribution, ANOVA for significance testing, Pearson correlation for data consistency, and a paired sample *t* test to compare Brixia scores between COVID-19 and H1N1 patients.

### 2.4. Ethical approval

Institutional review board approval was obtained from the Institutional Review Board, Jordan University of Science and Technology (#11/138/2021). A consent form was waived due to the retrospective study design.

## 3. Results

Table 1 shows baseline clinical characteristics and clinical outcomes of COVID-19 and H1N1 patients. More males were affected in both groups, however the proportion of COVID-19 cases (56.5%) was higher than H1N1 (53.2%). COVID-19 was more common in older adults (60+ years = 57.5%), whereas H1N1 was more common in younger individuals (0–40 years = 51.8%). Most patients in both groups were discharged within 15 days. Diabetes (48.2%) and hypertension (57.3%) were more prevalent in COVID-19 patients than in H1N1 patients (24.8% and 26.1%, respectively). The proportion of preexisting malignancies and lung disease was higher in H1N1 patients, whereas chronic kidney disease was more frequent in COVID-19 patients. Most patients required fewer than 5 X-rays. Complications, such as pneumothorax, pleural effusion, and pneumomediastinum, were rare in both groups, however, a slightly higher proportion was observed in COVID-19 patients.

**Table 1:** Baseline characteristics and clinical outcomes of COVID-19 and H1N1 patients.

Variable name	Components	COVID-19		H1N1	
		Frequencies	*f* %	Frequencies	*f* %
Gender	Male	538	56.5	118	53.2
	Female	415	43.5	104	46.8
Age	0–40	89	9.3	115	51.8
	41–50	109	11.4	23	10.4
	51–60	207	21.7	25	11.3
	60+	548	57.5	59	26.6
Time to discharge	1–15	786	82.5	192	86.5
	16–30	139	14.6	22	9.9
	31–45	23	2.4	5	2.2
	46–60	4	.4	2	.9
	60+	1	.1	1	.4
Diabetes mellitus	Absent	493	51.7	167	75.2
	Present	459	48.2	55	24.8
Hypertension	Absent	407	42.6	164	73.9
	Present	546	57.3	58	26.1
Preexisting malignancy	Absent	882	92.5	199	89.6
	Present	71	7.5	23	10.4
Chronic kidney disease	Absent	848	89.0	212	95.5
	Present	105	11.0	10	4.5
Lung disease	Absent	931	97.7	200	90.1
	Present	22	2.3	22	9.9
No. of X-rays	0–4	621	65.2	187	84.2
	5–10	171	17.9	17	7.7
	11–20	124	13.0	10	4.5
	21–30	28	2.9	6	2.7
	30+	9	.9	2	0.9
Pneumothorax	Absent	947	99.4	221	99.5
	Present	6	.6	1	.5
Pleural effusion	Absent	922	96.7	215	96.8
	Present	31	3.3	7	3.2
Pneumomediastinum	Absent	928	97.4	222	100.0
	Present	25	2.6	0.0	0

Table 2 shows descriptive statistics for important variables in COVID-19 and H1N1 patients. COVID-19 patients are older (mean age = 60.72) compared to H1N1 patients (mean age = 36.00). The time to discharge is higher in H1N1 patients (mean = 7.32 days) compared to COVID patients (mean = 1.21 days). COVID-19 patients have higher admission scores (mean = 8.35) and discharge scores (mean 12.64) than H1N1 patients (mean admission score = 6.77, discharge score = 12.33).

**Table 2:** Descriptive statistics for clinical and prognostic variables in COVID-19 and H1N1 patients.

	COVID-19					H1N1				
	Age	Survival and non-survival	Time to discharge	Score at admission	Score at discharge	Age	Survival and non-survival	Time to discharge	Score at admission	Score at discharge
Mean	60.72	0.29	1.21	8.35	48.44	36.00	0.16	7.32	6.77	12.33
Median	62.00	0.00	1.00	10.00	26.50	36.27	0.00	4.00	6.00	4.00
Std. deviation	0.994	0.455	0.503	7.237	75.29	1.298	0.369	0.555	4.602	28.43

Table 3 compares the discharge status (alive or deceased) of COVID-19 and H1N1 patients based on their Brixia X-ray scores at admission, their highest score, and how much their scores increased. For both diseases, higher increments in Brixia scores and highest score are associated with increased mortality. In COVID-19 patients, a large number of those with the highest scores (13–18) did not survive. In H1N1 patients, even though fewer patients had very high scores, mortality was still high.

**Table 3:** Cross-tabulation of the discharge status of COVID-19 and H1N1 patients with their Brixia X-ray scores at admission and highest score.

COVID-19					
		Score at admission			Total
		0–6	7–12	13–18	
Mortality	Alive	250	220	205	675
	Deceased	53	65	160	278
Total		303	285	365	953
		Highest Brixia score			Total
		0–6	7–12	13–18	
Mortality	Alive	388	89	197	674
	Deceased	54	27	197	278
Total		443	117	394	952
		Incremented score			Total
		-ve	0–2	3+	
Mortality	Alive	272	262	141	675
	Deceased	52	106	120	278
Total		324	368	261	953
** **H1N1					
		Score at admission			Total
		0–6	7–12	13–18	
Mortality	Alive	110	59	17	186
	Deceased	15	9	12	36
Total		125	68	29	222
		Highest Brixia score			Total
		0–6	7–12	13–18	
Mortality	Alive	138	32	16	186
	Deceased	12	3	21	36
Total		150	35	37	222
		Incremented score			Total
		-ve	0–2	3+	
Survival and non-survival	Alive	84	91	11	186
	Deceased	8	12	16	36
Total		92	103	27	222

* 0–18 = lowest to highest Brixia score.

The ANOVA results show significant differences in Brixia scores between discharged and deceased COVID-19 and H1N1 patients (*P* = .00) (Table 4). For COVID-19, the *F*-values were highest for the score at discharge/death (203.466), followed by the highest score recorded (55.477). Similar results were observed for H1N1, with the highest *F*-value for discharge/death scores (54.706) followed by the highest score recorded (49.728).

**Table 4:** Analysis of variance (ANOVA) to compare the discharge status of COVID-19 and H1N1 patients with their corresponding Brixia scores.

Score category	Sum of squares	df	Mean square	*F*-value	Sig.
COVID-19					
Score at admission					
Between groups	802.803	1	802.803	16.039	0.000
Within groups	16167.505	323	50.054		
Total	16970.308	324			
Highest score					
Between groups	2091.213	1	2091.213	55.477	0.000
Within groups	19488.224	517	37.695		
Total	21579.437	518			
Lowest score					
Between groups	673.332	1	673.332	36.826	0.000
Within groups	10037.862	549	18.284		
Total	10711.194	550			
Score at discharge/death					
Between groups	8549.593	1	8549.593	203.466	0.000
Within groups	36557.209	870	42.020		
Total	45106.803	871			
HIN1					
Score at admission					
Between groups	275.202	1	275.202	13.743	0.000
Within groups	4405.537	220	20.025		
Total	4680.739	221			
Highest score					
Between groups	1673.190	1	1673.190	49.728	0.000
Within groups	7402.238	220	33.647		
Total	9075.428	221			
Lowest score					
Between groups	594.595	1	594.595	38.414	0.000
Within groups	3405.279	220	15.479		
Total	3999.874	221			
Score at discharge/death					
Between groups	1391.466	1	1391.466	54.706	0.000
Within groups	5595.814	220	25.436		
Total	6987.279	221			

Table 5 shows the Pearson and partial correlation coefficients for Brixia X-ray scores in COVID-19 and H1N1 patients, controlling for various health conditions. In COVID-19 patients, all score comparisons show significant positive correlations (*P* = .00). The strongest correlation between admission and discharge/death scores (*R* = 0.616). The highest and lowest scores are also strongly correlated (*R* = 0.888). In contrast, the H1N1 patient data is incomplete. It shows only a moderate correlation (*R* = 0.572) between the highest score and the discharge/death score.

**Table 5:** Pearson and partial correlation to check consistency between Brixia X-ray scores on behalf of controlled variables in COVID-19 patients and H1N1 patients.

Control variables		Score at admission	Highest score	Lowest score	Score at discharge/death
COVID-19					
Gender & age & DM & HTN & malignancy & CKD & COPD or other lung dz & Ptx & lung fibrosis & effusion & pneumomediastinum	Score at admission	1.000	.310	.379	.616
		.	.000	.000	.000
	Highest score	.310	1.000	.888	.572
		.000	.	.000	.000
	Lowest score	.379	.888	1.000	.560
		.000	.000	.	.000
	Score at discharge/death	.616	.572	.560	1.000
		.000	.000	.000	.
H1N1 patients					
Gender & age & DM & HTN & malignancy & CKD & COPD or other lung dz & Ptx & lung fibrosis & effusion & pneumomediastinum	Score at admission	1.000			
		.			
	Highest score	.	1.000		
		.			
	Lowest score	.		1.000	
		.			.
	Score at discharge/death	.	.572	.560	1.000
		.	.	.	.

The paired samples *t* test indicates that COVID-19 patients had significantly higher Brixia scores at admission, peak severity, and lowest severity compared to H1N1 patients (*P* = .00). Scores at discharge or death were also significantly higher in COVID-19 patients (*P* = .00). Moreover, the difference in mortality status and score increment between the 2 groups was statistically significant (Table 6).

**Table 6:** Paired samples *t* test to compare Brixia X-ray scores with patient’s death status between COVID-19 and H1N1 patients.

		Paired differences					*t*	df	Sig. (2-tailed)
		Mean	Std. deviation	Std. error mean	95% Confidence interval of the difference				
					Lower	Upper			
Pair 1	Score at admission–score at admission	7.923	4.586	.899	-9.776	-6.071	-8.809	25	.000
Pair 2	Highest score–highest score	4.784	6.279	.730	-6.239	-3.329	-6.553	73	.000
Pair 3	Lowest score–lowest score	2.325	4.325	.484	-3.287	-1.363	-4.809	79	.000
Pair 4	Score at discharge/death–score at discharge/death	3.416	8.733	.622	2.189	4.643	5.491	196	.000
Pair 5	Death or not dead	.158	.592	.040	.079	.236	3.967	221	.000
Pair 6	Increment COVID-19–increment H1N1	1.87838	7.34284	.49282	.90715	2.84960	3.811	221	.000

Table 7 shows the model fit statistics for COVID-19 and H1N1. The Akaike Information Criterion (AIC) and Bayesian Information Criterion (BIC) values are higher for COVID-19 (AIC = 1205.042, BIC = 1214.759) than for H1N1 (AIC = 190.877, BIC = 197.682). The log-likelihood is lower for COVID-19 (-600.521) compared to H1N1 (-93.438). Pearson Chi-square values also show greater variance in COVID-19 than in H1N1.

**Table 7:** AIC score for model fit.

Test	Value
COVID-19	
Pearson Chi-square	196.819
Scaled Pearson Chi-square	952.000
Log Likelihood^b^	-600.521
Akaike Information Criterion (AIC)	1205.042
Bayesian Information Criterion (BIC)	1214.759
H1N1	
Pearson Chi-square	30.162
Scaled Pearson Chi-square	222.000
Log Likelihood^b^	-93.438
Akaike Information Criterion (AIC)	190.877
Bayesian Information Criterion (BIC)	197.682

The regression analysis using backward elimination shows that for COVID-19, the admission score, discharge/death score, and disease interaction increment have a significant impact on the model. In contrast, for H1N1, only the highest score is significant (Table 8).

**Table 8:** Regression using the backward elimination.

Model		Unstandardized coefficients				Standardized coefficients	t	Sig.
		B		Std. Error		Beta		
COVID-19								
1	(Constant)	-.049		.044			-1.115	.265
	Score at admission	.047		.020		.088	2.298	.022
	Score at discharge/death	.021		.003		.345	7.803	.000
	Increment (disease interaction)	.056		.022		.099	2.485	.013
H1N1								
1	(Constant)		-.149		.063		-2.386	.018
	Lowest score		.049		.072	.067	.681	.497
	Score at discharge/death		.029		.072	.053	.407	.684
	Score at admission		-.046		.048	-.089	-.956	.340
	Highest score		.177		.070	.368	2.537	.012
	Increment		.028		.051	.050	.542	.588

For COVID-19, a higher score at admission and the lowest score during the illness were significantly associated with the outcome. The lowest score has a stronger effect (Exp(B) = 1.664, *P* = .00). The highest score was not significant. As for H1N1, the highest score during the illness was the only significant predictor (Exp(B) = 4.174, *P* = .001) (Table 9). Other scores such as admission and discharge scores, were not significant. The constants for both models show a low baseline probability of the outcome.

**Table 9:** Scoring system predictability.

		B		SE	Wald	*d*f		Sig.	Exp(B)
COVID-19	Score at admission	.100		.038	6.952	1		.008	1.105
	Highest score	-.181		.258	.490	1		.484	.835
	Lowest score	.509		.095	28.476	1		.000	1.664
	Constant	-2.418		.256	89.468	1		.000	.089
H1N1	Score at admission		-.560	.409	1.879	1	.170		.571
	Score at discharge/death		.184	.477	.149	1	.699		1.202
	Highest score		1.429	.441	10.514	1	.001		4.174
	Lowest score		.264	.496	.284	1	.594		1.303
	Constant		-3.850	.555	48.154	1	.000		.021

## 4. Discussion

This study assessed the prognostic utility of the Brixia score in predicting outcomes in patients with COVID-19 and H1N1 viral infections. Our findings show that higher Brixia scores at admission, peak severity, and at discharge or death are significantly associated with increased mortality. COVID-19 patients had significantly higher Brixia scores in all phases of illness compared to H1N1 patients.

Borghesi and Maroldi^[14]^ initially proposed the Brixia score to quantify lung involvement in COVID-19, reporting its predictive value for mortality and disease severity. Similarly, Wu et al^[24]^ found that elevated Brixia scores were associated with higher mortality in COVID-19 patients. The present study expands on these findings by also including H1N1 patients and shows that, even though Brixia score is predictive for both infections, COVID-19 patients exhibit more severe radiographic findings, corroborating prior reports showing that ventilator-associated pneumonia occurs more frequently in COVID.^[25]^

The role of chest radiographic scoring in viral pneumonia extends beyond COVID-19. In the context of H1N1, Abbo et al^[26]^ examined imaging findings in H1N1 influenza and showed variable patterns of lung involvement. Our study shows that, even though the Brixia score retains its prognostic value in H1N1, the overall burden of radiographic abnormalities is lower compared to COVID-19 since the admission scores were lower.

Importantly, our regression analysis indicates that for COVID-19, the discharge/death Brixia scores were the most significant predictors of mortality. However, for H1N1, the highest Brixia score was the only significant predictor. This observation shows that the disease trajectory in H1N1 may be less progressive than in COVID-19. This observation is in line with the work done by Al Umairi et al,^[27]^ which shows that higher chest radiographic severity scores were more commonly associated with adverse outcomes in COVID-19 patients.

An important finding of our study is the correlation between Brixia score increments and mortality. Patients with greater increases in Brixia scores from admission to peak severity were more likely to succumb to their illness. This aligns with prior work by Kohli et al,^[28]^ who demonstrated that serial radiographic assessment can aid in patient monitoring when assessed alongside their clinical status in a hospital setting. Since H1N1 patients show lower increases in Brixia scores, our results further support the fact that COVID-19 presents a more progressive disease course with sustained lung involvement over time.

The clinical implications of these findings demonstrate the value of early and ongoing chest radiographic assessment in viral pneumonias. Even though other scales like the CO-RADS provide a well‑validated, categorical CT‑based framework for grading pulmonary involvement, demonstrating high performance in moderate to severe cases and strong interobserver consistency, especially at the extremes (categories 1 and 5), its reliance on CT limits its applicability in many settings.^[29]^ In comparison, the Brixia score provides a straightforward, cost-effective method for quantifying lung parenchymal changes using standard CXRs, which are far more accessible than CT in both established and resource‑constrained healthcare facilities.^[30]^ Unlike CO-RADS, which benefits from extensive validation but demands high-end imaging infrastructure, the Brixia system fills an important gap by delivering an objective, scalable severity index for CXRs. This makes it especially valuable during surges of respiratory infections, such as the COVID‑19 pandemic, when CT capacity may be overwhelmed or unavailable, and when rapid, repeatable assessments of disease progression are essential.

Even though this study provides valuable insights, it also has several limitations which should be acknowledged. First, the retrospective design introduces inherent biases, and prospective studies are needed to confirm these findings. Second, variability in observing radiological data by radiologists remains a potential confounder in radiographic scoring which also depends on their experience. Third, clinical parameters such as inflammatory biomarkers and oxygenation indices were not included in our analysis, which could further improve the predictive power of the Brixia score.

## 5. Conclusion

Our study demonstrates the prognostic value of the Brixia score in predicting outcomes across COVID-19 and H1N1 patients. COVID-19 patients had higher Brixia scores and greater score increments over time, correlating with increased mortality. These findings demonstrate the role of chest radiographic assessment in early risk determination and clinical decision-making.

## Author contributions

**Conceptualization:** Ruba Khasawneh, Basheer Khassawneh, Firas A. Khasawneh, Maha Gharaibeh, Sarah Athamneh, Tarek Ajam, Mohammad Z. Nofal.

**Data curation:** Ruba Khasawneh, Basheer Khassawneh, Sarah Athamneh, Tarek Ajam, Mohammad Z. Nofal.

**Formal analysis:** Ruba Khasawneh, Firas A. Khasawneh

**Investigation:** Ruba Khasawneh, Basheer Khasawneh, Sarah Athamneh, Tarek Ajam, Mohammad Z.Nofal.

**Methodology:** Ruba Khasawneh, Firas A. Khasawneh.

**Resources:** Ruba Khasawneh, Basheer Khassawneh.

**Supervision:** Ruba Khasawneh, Basheer Khassawneh, Firas A. Khasawneh, Maha Gharaibeh.

**Validation:** Ruba Khasawneh, Basheer Khassawneh, Firas A. Khasawneh, Maha Gharaibeh.

**Visualization:** Ruba Khasawneh, Firas A. Khasawneh, Sarah Athamneh, Tarek Ajam, Mohammad Z. Nofal.

**Writing – original draft:** Ruba Khasawneh, Firas A. Khasawneh.

**Writing – review & editing:** Ruba Khasawneh, Basheer Khassawneh, Firas A. Khasawneh, Maha Gharaibeh, Sarah Athamneh, Tarek Ajam, Mohammad Z. Nofal.
